# Spaceflight Modifies *Escherichia coli* Gene Expression in Response to Antibiotic Exposure and Reveals Role of Oxidative Stress Response

**DOI:** 10.3389/fmicb.2018.00310

**Published:** 2018-03-16

**Authors:** Thomas R. Aunins, Keesha E. Erickson, Nripesh Prasad, Shawn E. Levy, Angela Jones, Shristi Shrestha, Rick Mastracchio, Louis Stodieck, David Klaus, Luis Zea, Anushree Chatterjee

**Affiliations:** ^1^Department of Chemical and Biological Engineering, University of Colorado Boulder, Boulder, CO, United States; ^2^Genomic Services Laboratory, HudsonAlpha Institute for Biotechnology, Huntsville, AL, United States; ^3^Department of Biological Sciences, The University of Alabama in Huntsville, Huntsville, AL, United States; ^4^Astronaut Office, Johnson Space Center, National Aeronautics and Space Administration, Washington, DC, United States; ^5^BioServe Space Technologies, Department of Aerospace Engineering Sciences, University of Colorado Boulder, Boulder, CO, United States; ^6^Department of Aerospace Engineering Sciences, University of Colorado Boulder, Boulder, CO, United States; ^7^BioFrontiers Institute, University of Colorado Boulder, Boulder, CO, United States

**Keywords:** *Escherichia coli*, spaceflight, RNA-sequencing, antibiotic, tolerance, oxidative stress, bioastronautics, microgravity

## Abstract

Bacteria grown in space experiments under microgravity conditions have been found to undergo unique physiological responses, ranging from modified cell morphology and growth dynamics to a putative increased tolerance to antibiotics. A common theory for this behavior is the loss of gravity-driven convection processes in the orbital environment, resulting in both reduction of extracellular nutrient availability and the accumulation of bacterial byproducts near the cell. To further characterize the responses, this study investigated the transcriptomic response of *Escherichia coli* to both microgravity and antibiotic concentration. *E. coli* was grown aboard International Space Station in the presence of increasing concentrations of the antibiotic gentamicin with identical ground controls conducted on Earth. Here we show that within 49 h of being cultured, *E. coli* adapted to grow at higher antibiotic concentrations in space compared to Earth, and demonstrated consistent changes in expression of 63 genes in response to an increase in drug concentration in both environments, including specific responses related to oxidative stress and starvation response. Additionally, we find 50 stress-response genes upregulated in response to the microgravity when compared directly to the equivalent concentration in the ground control. We conclude that the increased antibiotic tolerance in microgravity may be attributed not only to diminished transport processes, but also to a resultant antibiotic cross-resistance response conferred by an overlapping effect of stress response genes. Our data suggest that direct stresses of nutrient starvation and acid-shock conveyed by the microgravity environment can incidentally upregulate stress response pathways related to antibiotic stress and in doing so contribute to the increased antibiotic stress tolerance observed for bacteria in space experiments. These results provide insights into the ability of bacteria to adapt under extreme stress conditions and potential strategies to prevent antimicrobial-resistance in space and on Earth.

## Introduction

Among the many risks astronauts will face as they venture into missions beyond lower Earth orbit are those that arise from microbial responses to spaceflight. Immune dysfunction associated with spaceflight conditions can potentially increase the susceptibility of crew members to pathogenic bacteria in extended space missions (Borchers et al., 2002; Sonnenfeld and Shearer, 2002). Simultaneously, bacteria introduced in a spaceflight environment inhabited by crew members have exhibited antibiotic-resistance traits, thus posing a threat to spaceflight missions. Spaceflight has been shown to promote biofilm formation in bacteria, which may pose challenges involving biofouling, corrosion, the contamination of water sources, and increased bacterial virulence (McLean et al., 2001; Kim et al., 2013; Ott et al., 2016). Changes of microbial behavior observed in space include improved growth (Zea et al., 2017), decreased susceptibility to antibiotics (Tixador et al., 1985; Lapchine et al., 1986; Moatti et al., 1986; Tixador et al., 1994; Klaus and Howard, 2006; Kitts et al., 2007; Parra et al., 2008; Ricco et al., 2010), enhanced capability to form biofilms (McLean et al., 2001; Kim et al., 2013), formation of outer membrane vesicles (Zea et al., 2017), and increased virulence (Wilson et al., 2007, 2008), to name a few. In the case of cultures grown in liquid medium, some of these responses may result from an altered extracellular environment in space in which mass transport is limited to diffusion due to the lack of gravity driven forces, as was recently corroborated by a molecular genetic study (Zea et al., 2016). At a time when understanding bacterial resistance mechanisms is increasingly important on Earth as multi-drug resistant strains have become increasingly common, experiments in microgravity offer another avenue through which antibiotic effectiveness may be explored (Erickson et al., 2015; Otoupal et al., 2017).

The wide variety and consistency of the altered stress responses reported in *Escherichia coli* is useful both for understanding the general adaptive mechanisms in the bacterial stress response as well as for understanding and explaining *E. coli*’s reduced sensitivity to antibiotics in microgravity. Furthermore, given the projected increase in space travel and exploration in the future, it is imperative that we better understand mechanisms that allow bacteria to thrive in presence of extreme stresses and prepare countermeasures. In fact the National Aeronautics and Space Administration (NASA) has identified several knowledge gaps that need to be addressed, including the extent to which current antimicrobial therapies are effective against microbes altered by spaceflight, efficacy of current countermeasures, and whether spaceflight induces changes in diversity, concentration, and/or characteristics of medically significant microorganisms which could affect crew health (Human Research Roadmap, 2017). To provide further insight to help answer these questions, in this study we investigated the changes in the transcriptome of *E. coli* cultured on the International Space Station (ISS) when challenged with different concentrations of the antibiotic gentamicin sulfate (an aminoglycoside that interrupts protein synthesis by binding to the 30S subunit of the bacterial ribosome), compared to controls grown on Earth.

**FIGURE 1:**
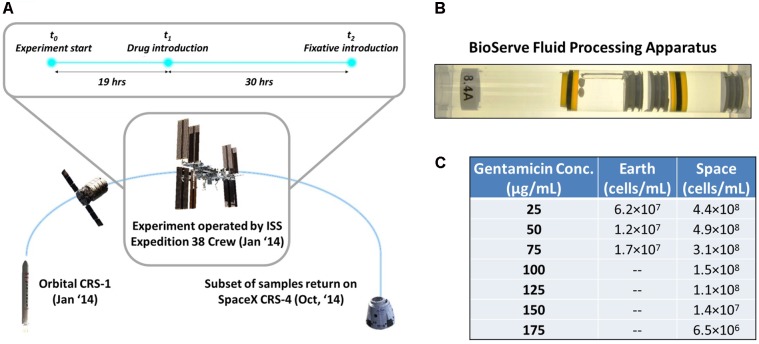
Microgravity experimental design and response of *E. coli* to antibiotic exposure in space and Earth. **(A)** The bacterial samples were launched aboard the Orbital CRS-1 mission and were carried up to the International Space Station, where they underwent a 49-h experiment consisting of a growth phase (19 h) and an antibiotic challenge phase (30 h), before a fixative was introduced. **(B)** By using rubber septa, the BioServe Fluid Processing Apparatus (FPA) was divided in four chambers. From left to right: growth medium with two Teflon balls to ensure mixing in microgravity (astronauts were requested to shake the hardware after each mixing), inoculum, antibiotic, fixative. Astronaut operations pushed all chambers from right to left, to enable the mixing of two consecutive chambers via the bypass (in this figure, observed over the growth medium chamber). **(C)** Cells/mL of surviving *E. coli* for each experimental condition upon introduction of antibiotic. Cells did not grow on Earth at higher than 75 μg/mL gentamicin concentration.

Several past investigations have included transcriptomic analyses to elucidate the governing molecular mechanisms behind increased bacterial tolerance to antibiotics. For example, Crabbé et al. (2011) concluded that certain virulence-related genes of *Pseudomonas aeruginosa* were induced in spaceflight and that the protein Hfq was a global transcriptional regulator. The latter had also been previously reported on *Salmonella typhimurium* by Wilson et al. (2007). Nevertheless, significant knowledge gaps remain to be addressed. This study describes the results of transcriptomic analyses that enabled determining the differentially expressed genes on samples at different gravitational regimes and concentrations of drug, and complements previous work (Zea et al., 2016, 2017) based on data from the same spaceflight experiment [Antibiotic Effectiveness in Space (AES-1)]. Specifically, this current study focuses on expression patterns and possible interaction of *E. coli* stress regulators associated primarily with oxidative and antibiotic stress, and examines how exposure to microgravity influences those regulatory interactions (**Figure 1A**). The genes identified could be useful for designing more effective antimicrobials both for space explorations as well as new drug options for Earth.

## Materials and Methods

### Experimental Design

The experiments were conducted using the non-pathogenic *E. coli* strain ATCC^®^ 4157^TM^, a non-motile strain that was chosen for assessing the transport-limiting properties of a microgravity environment. As *E. coli* is the most commonly studied bacteria in space, this choice maximized the ability to directly compare results against other experiments (Zea et al., 2014). *E. coli* ATCC^®^ 4157^TM^ was grown in Medium E minimal medium (Vogel and Bonner, 1956; Zea et al., 2014) and supplemented with glucose at a concentration of 27.8 mM (5 g/L). Growth in the ground controls took place in a temperature and humidity controlled environment designed to mimic the conditions aboard ISS during orbit. The experiment was designed for 30°C and was maintained close to that temperature, with space experiments being conducted at 30.2 ± 0.7°C and Earth experiments being conducted at 31.7 ± 0.4°C.

The antibiotic used in these experiments was gentamicin sulfate (MP Biomedical, Cat No. 1676045, Santa Ana, CA, United States), prepared such that concentrations would range from 0 to 75 μg/mL on Earth and 0 to 175 μg/mL in space, in 25 μg/mL increments. In order to be able to perform sample analysis on Earth of the data collected in space, the fixative RNALater II (Life Technologies, Cat No. B7024, Carlsbad, CA, United States) was used at a 0.6 fixative:sample volume ratio to terminate the experiment.

All samples were processed in BioServe’s Fluid Processing Apparatus (FPA) (Zea, 2015) in order to enable the experiment to take place in microgravity and use the same device for the controls (**Figure 1B**). The four different components of the experimental system—growth medium, inoculum, antibiotic, and fixative—were loaded into four separate compartments of the FPA, divided by septa that could be pushed through to allow mixing of the isolated components during the experiment. Forty-eight total FPAs were used to allow for 4 replicates each of the 12 experimental conditions.

The FPAs were loaded with 2.75 mL of the sterile Medium E with 5.91 g/L of glucose (eventually diluted to 5 g/L by time of experiment start) into chamber A of the FPA. After this initial loading the FPAs were incubated for 48 h and checked for contamination. Next, 0.5 mL of the *E. coli* inoculum was charged to chamber B in a glucose-free Medium E to ensure minimal growth prior to the start of the experiment (cell concentration after chambers A and B were mixed was 1.22 × 10^6^ cell/mL). The antibiotic solution in its respective concentration for each experiment was then added in 0.25 mL to chamber C of each FPA. Finally, 2.1 mL of the RNALater II fixative was added to chamber D of the FPAs. Full details of the experimental setup and design can be found in Zea (2015).

### Experimental Conditions

The experiment was conducted with two variables: concentration of the antibiotic gentamicin, with zero-concentration experiments as control, and gravitational regime, with 1g Earth experiments as control. Four experiments were conducted on Earth, with gentamicin concentrations of 0, 25, 50, and 75 μg/mL introduced to the cultures. Eight experiments were conducted in space, with 0, 25, 50, 75, 100, 125, 150, and 175 μg/mL of gentamicin, as it was anticipated that the space cultures would be able to survive in higher concentrations of the antibiotic. Four replicates were run for each of these conditions. The experiments for Earth and space were conducted asynchronously over the course of 49 h at the University of Colorado Boulder and onboard the ISS, respectively (**Figure 1A**). The inoculum was introduced to the growth medium at *t* = 0 h, followed by the introduction of gentamicin at *t* = 19 h. At *t* = 49 h the fixative was introduced to end the experiment. Unfortunately, for samples corresponding to 0 μg/mL on Earth and in space, the fixative was introduced prematurely, so these data could not be used for the analysis of gene expression, but did provide a starting inoculum cell count. Thus, gentamicin response was evaluated using differential expression of each condition relative to the lowest exposure at 25 μg/mL in their respective environment (Earth or space). This yielded comparison across eight conditions: two for Earth, six for space.

### Operations Timeline

After loading of the FPAs for both the Earth and space experiments with the growth media, inoculum, gentamicin solution, and fixative, the samples for the space experiments were introduced into BioServe’s Group Activation Pack (GAP), which in turn was placed in a Commercial Generic Bioprocessing Apparatus (CGBA) refrigerator/incubator. The CGBA was integrated into the Cygnus spacecraft (Orbital CRS-1 mission, **Figure 1A**), while the Earth samples were kept in the BioServe labs at the University of Colorado, Boulder. All samples were maintained at 4°C until the start of the experiment. After berthing to the Space Station, CGBA was transferred into ISS. CGBA was commanded to warm up to samples to 30°C and the inoculum was introduced into the growth medium. Nineteen hours after introduction of the inoculum, the antibiotic was also injected into the mix. Thirty hours after the introduction of the antibiotic, the RNALater II fixative was applied to end the experiment, summing to 49 total hours of active culture. All experiments were repeated on Earth to the exact timeline as in space, with an 8-h offset to ensure synchronization. The inactive culture tubes were then stored aboard ISS in a freezer at -80°C until the Dragon spacecraft (SpaceX CRS-4) returned from the ISS, 10 months later. Ground samples were also stored at -80°C during this time. Unfortunately, the 0 μg/mL samples on Earth and in space were not properly collected so the data from these experiments were not useful.

### RNA-Seq Data Preparation

Methods for RNA-Seq preparation are detailed previously by Zea et al. (2016). Briefly, bacterial suspension in RNALater II and Medium E growth medium were spun down, the supernatant was discarded, and the pellets were resuspended in PBS and mixed by pipetting. RNA was extracted with the Qiagen RNeasy mini kit (Qiagen, Hilden, Germany) with on-column DNase digestion. Integrity and concentration of the total RNA was estimated using the Agilent 2100 Bioanalyzer (Applied Biosystems, Carlsbad, CA, United States) and the Quant-iT^TM^ RiboGreen1 RNA Assay Kit (Thermo Fisher Scientific, Waltham, MA, United States), respectively. Ribosomal RNA (rRNA) was removed using Ribo-Zero^TM^ Gold (Yeast) kit (Epicenter, Madison, WI, United States). After removal of the rRNA, the RNA was fragmented and primed for the first strand synthesis using the NEBNext First Strand synthesis module (New England BioLabs Inc., Ipswich, MA, United States). The second strand synthesis was performed next with the NEBNext Second Strand synthesis module. Post-processing was done for the sequencing reads from RNA-seq experiments using the Hudson Alpha data analysis pipeline.

The FASTQ files were run through the Trimmomatic program to remove Illumina adapters corresponding to the TruSeq3 paired-end adapter library, as well as low-quality reads (below a quality score of 3) as well as bases within a 4-base sliding window in which the average base quality score dropped below 15. Additionally, reads shorter than 36 bases were dropped.

### Differential Gene Expression Analysis

The effect of gentamicin and microgravity on the *E. coli* cultures was assessed by measuring whether genes are differentially expressed (DE) and differentially variable (DV) between different experimental conditions. The *E. coli* NCTC 86 genome assembly was obtained from the NCBI RefSeq database^1^ and indexed for mapping using Bowtie2 (Langmead et al., 2009). The trimmed paired-end FASTQ read files were mapped to this reference genome using Bowtie2 with the “very sensitive” alignment condition, and the output SAM files for this were converted to sorted BAM files for RNA-seq. The RNA-seq alignment of each replicate was examined for alignment percentage, and alignment of 20% was used as a cutoff for eliminating low scores from our analysis. This left 3 replicates for each of the experimental conditions, except for the Earth 25 μg/mL and space 175 μg/mL (4 replicates each), and the space 150 μg/mL (2 replicates). Count tables were prepared from the sorted BAM files using the HTSeq Python package, and these were then analyzed for differential expression using the DESeq R package, which accounts for large data sets using the Holm-Bonferroni method.

### Differential Gene Expression Variability Analysis

Differential variability was calculated by normalizing the count tables output, corresponding to gene expression levels for each gene in each replicate, from the HTSeq script by using the sizeFactors function within the DESeq R package. The normalized count tables were then used to calculate average and standard deviation of gene expression across all four replicates for each antibiotic concentration, from which the coefficients of variation could be determined. For the space versus Earth comparison all experimental conditions in each setting were used to calculate a pooled variance for each gene on space and on Earth. This requires the assumption of equal variance across all genes within a given setting, which was taken to be reasonable based on the results of the differential variability analyses across gentamicin concentrations. As with the differential expression calculation, the Holm-Bonferroni method was used to address the problem of multiple comparisons.

## Results

### *E. coli* Response to Increasing Gentamicin Concentration

In these experiments it was found that *E. coli* cultures grown in the presence of gentamicin in microgravity were able to survive at higher concentrations of the drug than in a 1g gravitational regime on Earth (**Figure 1C**). To explore this phenomenon we have examined transcriptomic data from 7 experiments run in space, and 3 on Earth, each at a different concentration of gentamicin (see section “Materials and Methods”).

**FIGURE 2:**
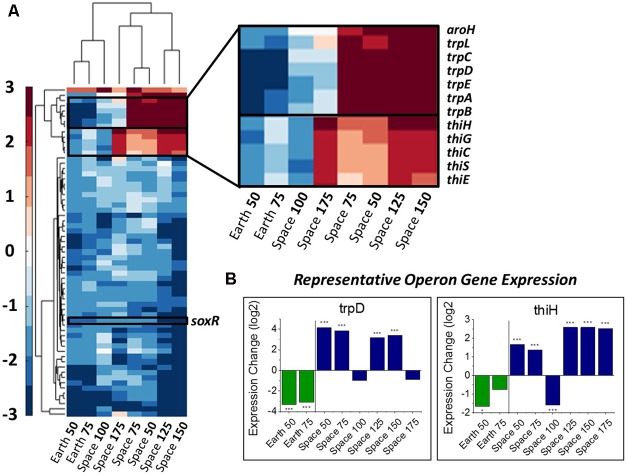
Overall gene response to gentamicin and the *trp* and *thi* operons. **(A)** Heat map of the 63 genes that show differential expression consistently with respect to increasing gentamicin concentration (complete heat map with genes in **Supporting Information**). Emphasis on genes with differing response on Earth versus in space. **(B)** Representative expression patterns in two representative genes from the *trp* and *thi* operons. Expression change is measured in log2 fold change (^∗^*P*-value < 0.05, ^∗∗∗^*P*-value < 0.001).

Differences in gene expression with respect to drug concentration were calculated by comparing the expression levels for a gene for each experiment relative to the 25 μg/mL experiment in their respective environment as a basis of comparison (the 0 μg/mL experiments had to be excluded, see section “Materials and Methods”). An alpha-value of 0.05 to represent significant levels of differential expression. We define a consistent gentamicin response to be significant differential expression in at least one comparison on Earth and at least four comparisons in space. Using this criterion, we identified 63 DE genes, whose expression profiles are shown in **Figure 2A**, along with a clustering of experimental conditions by similarity of expression pattern. The clustering of the space 100 μg/mL experiment with the two Earth conditions rather than the other space conditions demonstrates its incongruity with the rest of the space data, though it is not clear what might have caused this differing expression profile. For this reason, we avoid drawing conclusions based on its behavior. It should be noted that for 62 out of these 63 genes the 100 μg/mL space experiment is downregulated with respect to the 25 μg/mL experiment, which is what caused it to be clustered with the Earth experiments.

#### The *thi* and *trp* Operons Involved in Metabolism Show Differing Behavior in Space versus on Earth

In this set of 63 genes, genes of the *thi* and *trp* operons show opposite regulation patterns with respect to increasing gentamicin concentration in space versus on Earth. Moreover, the thiamine and tryptophan biosynthetic processes, involving genes of the *thi* and *trp* operons, were found to be overrepresented among the set of 63 DE genes in space, suggesting that they are particularly important to the gentamicin response (**Figure 2A**; Mi et al., 2017). The five *thi* genes and six *trp* genes show consistent expression profiles within their respective operons (**Figure 2B**). Another gene *aroH*, which is associated with the synthesis of aromatic amino acids such as tryptophan, was also found to be consistently DE in the pattern of the *trp* operon with respect to increasing drug concentration. Though the regulation patterns within these two operons appear similar when compared to their respective 25 μg/mL experiment, they differ when the Earth and space experiments at the three matched concentration levels (e.g., space 50 μg/mL vs. Earth 50 μg/mL) are compared (**Table 1**). The *thi* operon shows upregulation in space across all three matched experiments, whereas the *trp* operon shows downregulation in space at 25 μg/mL and upregulation at 50 and 75 μg/mL. As noted in a previous paper examining data from these experiments, the *thi* operon is associated with a nutrient starvation response, which is induced by bacterial growth in microgravity environments (Zea, 2015; Zea et al., 2016). Additionally, the gene *thiH* has also been linked to *E. coli*’s DNA damage stress response (Khil and Camerini-Otero, 2002).

**Table 1:** Comparison of key operon behavior in matched concentration experiments.

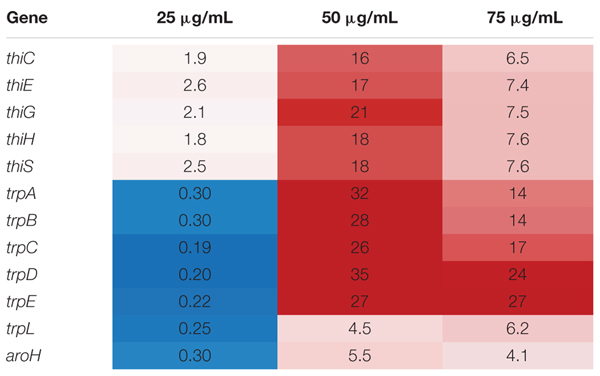

**The average expression level of a gene in an operon in the space experiment divided by the respective Earth experiment is shown. Blue corresponds to downregulation in space; red corresponds to upregulation in space.**

Similarly, the *trp* operon is associated with general amino acid synthesis, and may be related to an amino acid starvation response. These genes are also linked to the metabolism and production of the signaling molecule indole (Dunn et al., 1990; Yee et al., 1996), which has been shown to convey drug resistance, oxidative stress resistance, and acid resistance, and to improve virulence, plasmid stability and overall persistence in *E. coli* (Hirakawa et al., 2005; Lee and Lee, 2010; Han et al., 2011; Vega et al., 2012). Indole production responds to multiple environmental factors, including induction by bactericidal antibiotics such as gentamicin as well as repression by glucose availability and low pH (Han et al., 2011). However, the other operon involved in indole production, *tnaAB* (Han et al., 2011; Hu et al., 2015), was not found to be consistently or significantly up or downregulated with increasing gentamicin concentration.

#### The Oxidative, Antibiotic and General Stress Regulators Show Conflicting Responses to Gentamicin Increase

**FIGURE 3:**
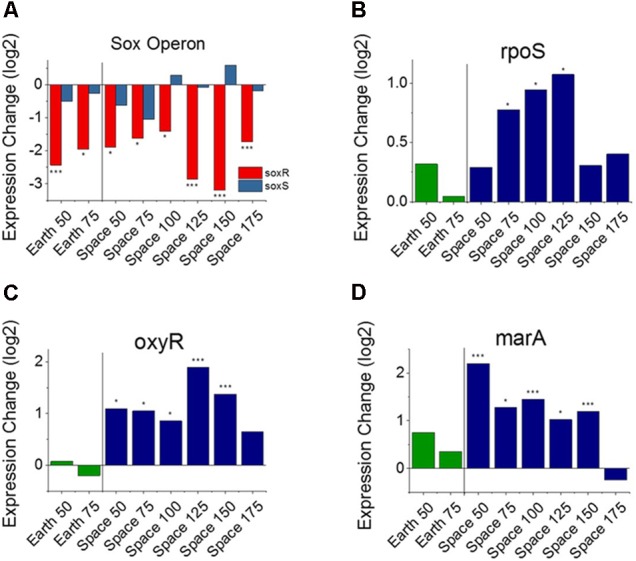
Oxidative stress response versus antibiotic concentration. Comparison of *soxR* and *soxS* **(A)**, shows conflicting regulation patterns relative to the 25 μg/mL experiments, as *soxR* is known to control transcription of *soxS*. Expression patterns of *rpoS* **(B)**, *oxyR* **(C)**, and *marA* **(D)** relative to the 25 μg/mL experiments on Earth and in space show consistent upregulation of oxidative stress response in space independent of the *soxRS* operon. Expression change is measured in log2 fold change (^∗^*P*-value < 0.05, ^∗∗∗^*P*-value < 0.001).

From the heat map in **Figure 2A**, we observe that nine genes involved with the *E. coli* stress response are consistently differentially expressed with respect to an increase in gentamicin. Five of these nine are specifically associated with oxidative stress (GO:0006979): *wrbA, uspE, ahpC, uspF*, and *soxR*. Though it has been shown that gentamicin induces an oxidative stress response in *E. coli* (Dwyer et al., 2014), we find all five of these oxidative stress response genes to be downregulated as gentamicin concentration increases. One gene that stands out in this set of stress response genes is *soxR* (labeled in **Figure 2A**), a broad regulator of superoxide stress response in *E. coli*. SoxR protein is activated through oxidation by redox-cycling antibiotics (Chiang and Schellhorn, 2012), and this oxidized form activates the gene *soxS*, which in turn activates numerous oxidative stress response genes. Despite consistent downregulation of *soxR*, however, no differential expression was found for *soxS* in any of the eight comparisons (**Figure 3A**). The only other genes that *soxR* is known to be able to regulate independent of *soxS*, *fumC*, and *acnA* (Fuentes et al., 2001), are DE in only one and two out of eight comparisons, respectively. This suggests that *soxR* repression occurs independently of any effects on downstream oxidative stress pathways. Consistent with this, the other four downregulated oxidative stress genes are not part of the *soxS* regulon.

In addition to the presence of oxidative species within the cell, three genes have been previously identified that may have a role in controlling or repressing the expression of *soxR*: *acrR*, *fur*, and *fnr* (Kumar and Shimizu, 2011; Lee et al., 2014). We found that *acrR* and *fur* did not show consistent differential expression across experimental comparisons. The gene *fnr*, which is responsible for negatively regulating *soxR*, was significantly upregulated in four of the six space comparisons (**Supporting Figure S1**), though this may not necessarily correspond to higher intracellular quantities of the FNR protein (Mettert and Kiley, 2005).

These findings with respect to oxidative stress response, led us to investigate other genes known to be major regulators of stress response in *E. coli*. In addition to the *soxRS* system, there are two other global regulators known to be responsible for oxidative stress in *E. coli* including *oxyR* and *rpoS* (Greenberg and Demple, 1989; Farr and Kogoma, 1991; Lynch et al., 2004; Wilson et al., 2007; Chiang and Schellhorn, 2012). OxyR protein is responsible for hydrogen peroxide resistance in *E. coli*, whereas *rpoS* is a general stress response gene whose regulon contains several genes involved in oxidative stress. *rpoS* was found to be overexpressed in 3 of the 6 space comparisons, a notable result which may indicate that a sustained effect exists (**Figure 3B**). *oxyR*, on the other hand, demonstrates significant upregulation in 5 of the 6 space comparisons, and none of the Earth comparisons (**Figure 3C**). Interestingly, when examining the genes that are regulated by *soxS* and *oxyR*, their expression patterns are the opposite of what we would expect. While *soxS* shows no increase in expression with increasing gentamicin, the eight genes that *soxS* regulates that are consistently DE are all upregulated at higher concentrations. Conversely, the seven genes that are regulated by *oxyR* and are consistently DE are all downregulated at higher concentrations (**Supporting Table S1**).

This result suggests that, at least in the space experiments, there is a complex regulatory response to an increase in gentamicin concentration from the oxidative stress resistance system. To explore this further, we examined operons that were consistently DE across the space experiments.

#### Stress Response Regulator Expression Patterns Indicate an Influence of Microgravity on Activation of Gentamicin Response

**FIGURE 4:**
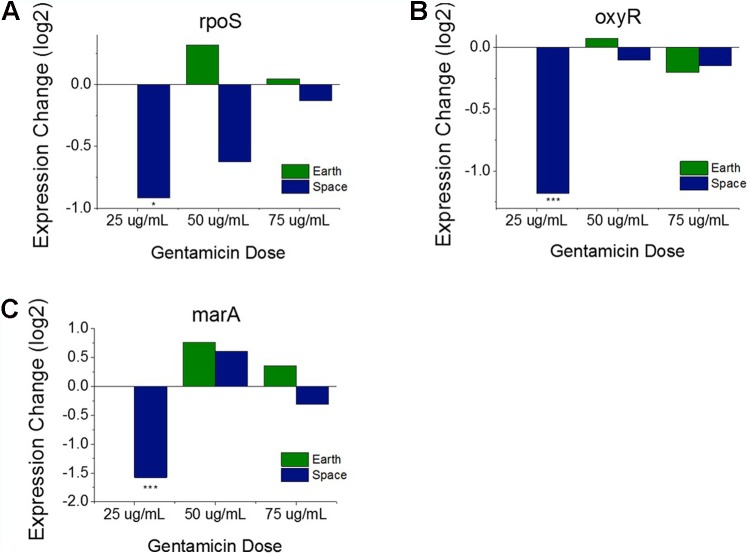
Oxidative stress response genes in matched drug concentration experiments. Expression levels of *rpoS* **(A)**, *oxyR* **(B)**, and *marA* **(C)** relative to respective Earth 25 μg/mL experiment shows a consistent expression pattern for all three genes. The 25 μg/mL experiment comparison is the only one to show significant differential expression. Expression change is measured in log2 fold change (^∗^*P*-value < 0.05, ^∗∗∗^*P*-value < 0.001).

Operons of interest were identified as those with at least two genes that are DE in at least five of the six space comparisons. This analysis identified 63 individual genes in 19 operons. Among the operons identified in this analysis is the *mar* operon, one of the broad primary regulators of antibiotic and oxidative stress response in *E. coli*. The gene *marA*, the primary transcriptional regulator of this operon, was found to be upregulated in five out the six space experiment comparisons, which is consistent with the expected response from increasing antibiotic concentration (**Figure 3D**). This stress gene, along with *rpoS* and *oxyR*, shows an elevated response with increasing gentamicin concentration in space and not on Earth, which is likely caused by differences in the basis of comparison: the 25 μg/mL drug concentration experiment in each respective environment. When matched space and Earth concentration experiments are compared for these genes, significant differences in expression are only observed at 25 μg/mL (**Figure 4**), with the space expression downregulated in each case. This provides evidence that the Earth experiments respond more strongly to antibiotic stress at 25 μg/mL than the space experiments.

### *E. coli* Response to Microgravity

#### *E. coli* Shows a Broadly Upregulated General Stress Response in the Microgravity Environment

**FIGURE 5:**
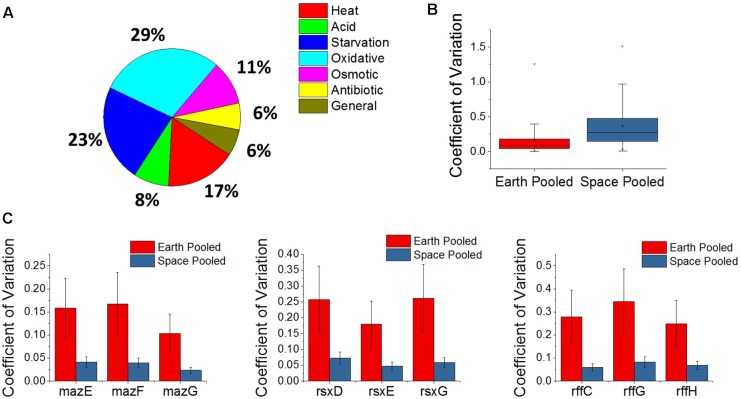
Analysis of the microgravity stress response and differential variability. **(A)** A pie chart distribution of stress response genes that are differentially expressed between space and Earth in matched experiments shows a broad assortment of stressors. Every differentially expressed stress response gene in this analysis is upregulated in space. **(B)** Box plot distributions for the coefficient of variation of expression of genes pooled across space or Earth in comparisons where one environment showed significantly different variability. The plot shows an overwhelming tendency for gene expression to be more variable in space. **(C)** Plots for the pooled coefficients of variation for each gene in operons that were less variable in space.

The *E. coli* response to microgravity was evaluated by examining the three matched space and Earth experiments at the gentamicin concentrations 25, 50, and 75 μg/mL for gene differential expression. A total of 109 genes were found to be DE in the same direction—all upregulated or all downregulated across all three of these comparisons. Within this set of genes, the GO classes for the TCA cycle, including the oxidation-reduction process (GO:0055114), were overrepresented (Mi et al., 2017). Fifty of these genes were found to be related to a stress response within *E. coli*, and show oxidative, starvation, and heat stresses as the most common (**Figure 5A**). All fifty stress response genes were overexpressed in space, indicating an increased stress response in all cases. Interestingly, we find this stress gene upregulation is in direct contrast with the expression patterns of the global regulators shown in **Figure 4**. Similar unstressed or underexpression behavior is also observed in global regulators *soxR* and *hfq* when comparing space experiments to Earth experiments.

#### Genes Show Overwhelmingly Higher Variability in Space Both in Response to Microgravity and Gentamicin

A new approach to analyze gene expression data involves measuring differences in gene expression variability across experimental conditions, and has been useful in examining gene interactions, behavior of human disease, and, most relevant to this study, bacterial adaptation (Ho et al., 2008; Mar et al., 2011; Park and Lehner, 2014; Erickson et al., 2017). Variability of gene expression across experiments was compared using the coefficient of variation, which is calculated by normalizing the standard deviation of gene expression level to the gene’s mean expression level, and pooling the space and Earth conditions for each gene (see section “Materials and Methods”). An *F*-test for equality of variance was then performed with a threshold *P*-value of 0.1 set for significant differential variability. Overall, it was found that the space experiments tended to show greater variation in gene expression across the four replicates of each condition than the Earth experiments, for individual genes (**Figure 5B**). Of the 5,406 genes examined, 1,980 of those were found to be differentially variable between Earth and space experiments. All but 28 of these were more variable in the space experiments. The overwhelmingly higher variability in space may indicate that *E. coli* under microgravity conditions can perform a wider search of transcriptome profiles under the less familiar stress condition of antibiotic, combined with effects due to microgravity. Additionally, the 28 genes that are more tightly regulated in space may represent a controlled response that the cell must adopt in response to microgravity. Of these 28 genes, three operons appeared multiple times (3 each): the *maz* operon, the *rff* operon, and the *rsx* operon (**Figure 5C**). None of these nine genes were consistently differentially expressed in the matched comparisons or across increasing gentamicin concentrations.

The *maz* operon encodes the MazEF toxin-antitoxin system which is involved in a wide variety of stress responses, inducing cell death to promote persister cell formation as well as a quasi-dormant state that is highly resistant to antibiotics (Suzuki et al., 2005). This is the second time we find a toxin-antitoxin system involved in the microgravity response, with the entericidin toxin-encoding gene *ecnB* showing greater than 19-fold upregulation in all three matched comparisons. These observations appear to indicate a role for toxin-antitoxin systems in mediating persister cell formation when the bacteria are stressed by microgravity. The *rff* operon encodes the enterobacterial common antigen, which is present on the surface of the bacteria family Enterobacteriaceae. The *rsx* operon encodes a system for the reduction of SoxR protein after it has been activated by an oxidizing species, though the lower variability of this operon in space does not explain the confounding behavior of *soxR* in the gentamicin differential expression comparisons.

## Discussion

### Expression of the *thi* and *trp* Operons

The analysis of effective gentamicin concentration gives clues to the differing roles of *thi* and *trp* operons in the *E. coli* gentamicin response. The *trp* operon shows a similar regulation pattern to *rpoS*, *oxyR*, and *marA* (**Figure 4**), with downregulation in the 25 μg/mL comparison contrasted to its upregulation in 50 and 75 μg/mL comparisons. In contrast, *thi* shows upregulation across all matched comparisons. This suggests that the *trp* operon is more closely related to the gentamicin response, whereas *thi* is likely more closely related to the microgravity response, similar to the 50 genes represented by the pie chart in **Figure 4A**. Indeed, the gene *thiH* is one of these 50 stress response genes. This is consistent with the *thi* operon being related to nutrient starvation (Zea et al., 2016) and the *trp* operon being associated with indole metabolism, a molecule that is associated with antibiotic resistance in *E. coli* (Dunn et al., 1990; Yee et al., 1996; Hirakawa et al., 2005; Lee and Lee, 2010; Han et al., 2011; Vega et al., 2012). It is notable that indole has shown that it can be repressed by low pH and high glucose availability, conditions specific to the space and Earth conditions, respectively (Han et al., 2011). However, without further data on cellular protein levels or with more independent controls, it is difficult to draw distinct conclusions from this expression behavior.

### Toxin-Antitoxin Systems for Creating Persister Cells

Through differential variability and matched concentration differential expression analysis, two instances of toxin-antitoxin systems were identified as possibly contributing to the difference in *E. coli’s* response to antibiotic in space versus on Earth. For the *mazEF* toxin system both the toxin (*mazE*) and the antitoxin (*mazF*) are less variable in the pooled space condition than the pooled Earth condition, though neither shows any consistent differential expression. This suggests that tight control of the toxin system balance is important for this irregular stress combination. Additionally, there is the toxin *ecnB*, which shows large overexpression in every matched drug concentration comparison. Its antitoxin, *ecnA*, shows differential expression only in the 25 μg/mL comparison, in which it is downregulated. This points to an important role of this entericidin system during microgravity response, which is consistent with past studies that have shown that *ecnB* is activated as a starvation response for forced apoptosis (Bishop et al., 1998). Together with the behavior *mazEF* system, it appears that mediating the cell-death and toxicity response is key for the bacteria to respond to antibiotics and microgravity in combination.

### Expression of Oxidative Stress Genes

Gentamicin has been shown to induce reactive oxygen species in its killing of bacterial cells (Sha and Schacht, 1999; Dwyer et al., 2014), leading us to hypothesize that an increasing gentamicin concentration in our experiments would cause higher expression levels of genes associated with oxidative stress. However, we observe a mix of responses among oxidative stress genes with respect to increases in antibiotic concentrations. The *marA* and *oxyR* genes, two of the broadest regulators of antibiotic and oxidative stress response in *E. coli* show expected behavior, increasing their expression from lower to higher gentamicin concentration in space. As discussed earlier, these genes do not show differential expression within the Earth comparisons, likely because the stress response is already activated at the 25 μg/mL condition and cannot be further upregulated. However, while the *marA* regulon is similarly upregulated with increasing gentamicin concentration, the *oxyR* regulon is consistently downregulated in genes that show significant differential expression. Further complicating the picture of oxidative stress response within these *E. coli* cultures is the expression of the *soxRS* oxidative stress system. The *soxR* gene is a global regulator of superoxide stress, and was found to be consistently downregulated in every comparison—both Earth and space—between the higher gentamicin concentrations and the 25 μg/mL concentration experiments. The *soxR* gene works by activating the transcription of *soxS*, which, in contrast, was not differentially expressed in any of the gentamicin comparisons. The *soxS* regulon was found to be consistently upregulated in the genes that show significant differential expression, which is likely caused by its large overlap with the *marA* regulon. This combination of expected and unexpected gene expression results suggests a complicated network of regulation.

The stress response genes *marA*, *oxyR*, and *soxR* are each induced by a separate signal within bacterial cells. The *marRAB* operon is activated by the presence of salicylate or a derivative aromatic acid, whereas the *oxyRS* and *soxRS* responses are primarily activated by the oxidative species H_2_O_2_ and superoxide, respectively (Martin et al., 2002; Gu and Imlay, 2011; Chiang and Schellhorn, 2012). Furthermore, it has been shown that the *soxS* gene is repressed by the presence of H_2_O_2_ at a concentration of 10 μM (Dwyer et al., 2014), orders of magnitude above the typical physiological concentration (Gonzalez-Flecha and Demple, 1997). In the same study, a 5 μg/mL concentration of gentamicin was found to increase expression of *soxS* and *oxyS*. These observations seem to point to H_2_O_2_ being the primary agent of oxidative stress in our system, as that would explain the simultaneous activation of the *oxyRS* operon and repression of the *soxRS* operon, but this still does not resolve the question of *oxyR* regulon downregulation or gentamicin’s demonstrated stimulation of *soxRS* expression.

Some of this unexpected regulation patterns may be attributed to interfering gene expression profiles caused by microgravity. A previous study of stress responses in low-shear modeled microgravity with *S. enterica* showed that the cells had a higher sensitivity to oxidative stress by H_2_O_2_ in the simulated microgravity environment (Wilson et al., 2002). However, while this effect is notable when examining the expression profile of our *E. coli* system, it does not necessarily agree with our analysis of differential gene expression in the matched concentration experiments. It may be useful in future studies for experiments to specifically monitor the transcription of these oxidative stress regulators, as well as their regulons, and to monitor the levels of their associated proteins over multiple experimental conditions.

### Effective Antibiotic Dose and Stress Response Overlap in Microgravity

We have shown evidence that the space experiments experience a lower effective dose of antibiotic than their matched Earth experiments. The diminished response of the genes *rpoS*, *oxyR*, and *marA*, each of which has been implicated in antibiotic defense, in the space 25 μg/mL experiments suggests that the stress response to gentamicin is only active at this concentration on Earth. However, when a higher concentration is introduced in space (50 and 75 μg/mL experiments), this response is activated. It has been established that in microgravity, extracellular mass transport is limited to diffusion in the absence of gravity-dependent forces, which would cause a lower effective dose of antibiotic to reach the cell in the same bulk concentration (Bjorkman, 1988; Albrecht-Buehler, 1991; Klaus et al., 1997; Brown et al., 2002; Zea et al., 2016). This conclusion is also supported via analysis of the morphological phenotypes presented by the cells in this experiment, as reported recently by Zea et al. (2017), which shows a minimal antibiotic effect at the space 25 μg/mL condition. This helps to explain one way that bacteria can survive at such high concentrations in space: in all experiments the effective dose of gentamicin in space is lower than at the same concentration on Earth.

In spite of this, however, we still observe consistent upregulation of 14 antibiotic and oxidative stress-related genes in a full comparison of the matched space experiments. One explanation for the upregulation of these genes may involve the overlap of stress response pathways controlled by broad regulators, where stress responses related to microgravity incidentally upregulate a wider variety of stress responses outside of the influence of the broad regulators we have explored. Numerous studies have established that the induction of both general and specific stress responses in *E. coli* can result in a broad resistance to other unrelated environmental stresses (Jenkins et al., 1988; Pichereau et al., 2000; Hengge-Aronis, 2002; Wilson et al., 2002; Nickerson et al., 2003). The global regulators *hfq* and *rpoS* have consistently been shown to participate in cross-resistance to antibiotics induced by microgravity, starvation, acid shock, or some combination of these stresses (Jenkins et al., 1988; McCann et al., 1991; Pichereau et al., 2000; Hengge-Aronis, 2002; Wilson et al., 2002; Lynch et al., 2004; Maurer et al., 2005).

These observations lead us to conclude that the microgravity environment, with its combination of acid and starvation stresses, likely conveys a wide array of environmental stress resistance. The higher tolerance of *E. coli* in microgravity to gentamicin is most likely due to a combination of two factors: (1) the lower effective dose of antibiotic reaching the cell due to transport limitations, and (2) the cross-resistance conveyed by the cell’s response to the acidic and nutrient-starved local environment in microgravity, a secondary consequence of reduced transport.

## Conclusion

Through the analysis of RNAseq data for *E. coli* challenged by both a microgravity environment and varying concentrations of the antibiotic gentamicin, we note several key observations that contribute to the picture of bacterial adaptive response to antibiotics, and provide new questions going forward in future research. Central to this paper is the response of oxidative stress regulators to increasing drug concentration in both gravitational regimes, as we note the counterintuitive regulation pattern of the superoxide response system *soxRS*, as well as the contradictory regulation patterns of the *oxyR* and *soxS* genes and their respective regulons. Further, we identify two toxin-antitoxin system genes, as well as the signaling molecule indole, which likely have a significant role in the adaptation of bacteria in space to the unique combination of stress it is experiencing. More research into exactly how these systems are contributing could help to fill in missing pieces in the understanding of bacterial resistance. Finally, we observe a universal increase in stress response in space experiments when compared to Earth experiments at the same concentrations of gentamicin. This provides substantial evidence for a cross-over of stress responses that helps to explain why bacteria are able to survive at such high concentrations of antibiotic in space, when they die at the same concentrations on Earth. The data and conclusions presented by this study pose a series of questions regarding the complex stress regulator networks in *E. coli* that can be probed in the future to help discern just how microgravity is affecting bacterial responses to drugs. Additionally, we believe that by building on these findings we will be able to provide new insights for the design of antibiotics that perform effectively in a variety of different environmental conditions.

## Author Contributions

TA: data analysis, manuscript draft writing, and editing. KE: data analysis and manuscript editing. NP, SL, AJ, SS, RM, LS, and DK: data collection and preparation. LZ: experimental design, supervision, and manuscript editing. AC: data analysis, manuscript draft writing, supervision, and manuscript editing.

## Conflict of Interest Statement

The authors declare that the research was conducted in the absence of any commercial or financial relationships that could be construed as a potential conflict of interest.

## Footnotes

1 ftp://ftp.ncbi.nlm.nih.gov/genomes/refseq

## Supplementary Material

**FIGURE S1 |** fnr expression pattern with respect to increasing gentamicin concentration. Expression of the fnr gene at various concentrations is compared to the 25 μg/mL concentration experiment in each respective environment (^∗^*P*-value < 0.05, ^∗∗∗^*P*-value < 0.001).

**TABLE S1 |** Expression patterns for the regulons of stress genes *soxS*, *oxyR*, and *marA* for space experiments with respect to 25 μg/mL concentration in space, for genes that show consistent differential expression (DE in 4+ comparisons). None of these genes showed mixed regulation, i.e., up and downregulation in separate comparisons. The column “# Space DE” indicates in how many comparisons in space significant differential expression was identified.
