# Supplementary figures and images for: Spaceflight Modifies Escherichia coli Gene Expression in Response to Antibiotic Exposure and Reveals Role of Oxidative Stress Response
Source: Front Microbiol. 2018 Mar 16;9:310. doi: 10.3389/fmicb.2018.00310 (PMC5865062; doi:10.3389/fmicb.2018.00310)

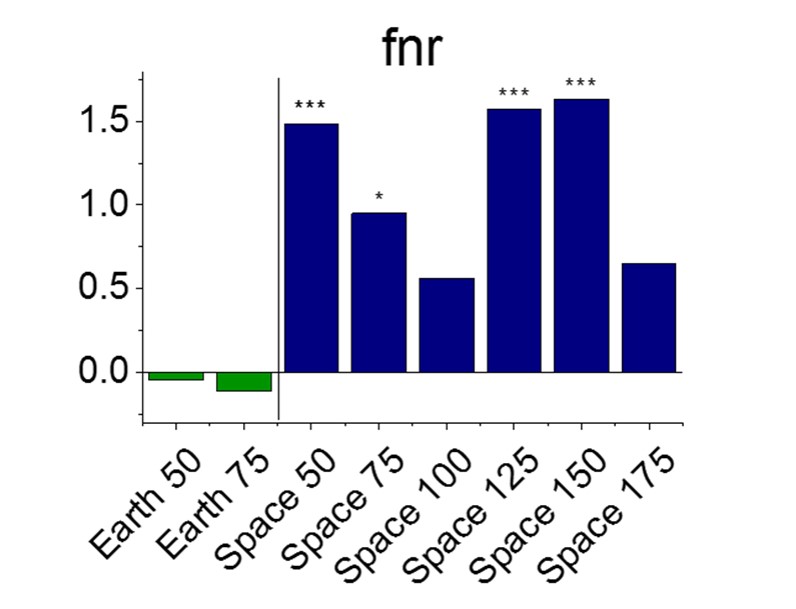

Supplement: Supplementary file 1 [file Image_1.JPEG]
