# Supplementary material for: Spaceflight Modifies Escherichia coli Gene Expression in Response to Antibiotic Exposure and Reveals Role of Oxidative Stress Response
Source: Front Microbiol. 2018 Mar 16;9:310. doi: 10.3389/fmicb.2018.00310 (PMC5865062; doi:10.3389/fmicb.2018.00310)
Supplement: Supplementary file 2 [file Table_1.DOCX]

**Supporting Table S1** Expression patterns for the regulons of stress genes *soxS*, *oxyR*, and *marA* for space experiments with respect to 25 µg/mL concentration in space, for genes that show consistent differential expression (DE in 4+ comparisons). None of these genes showed mixed regulation, i.e. up and downregulation in separate comparisons. The column “# Space DE” indicates in how many comparisons in space significant differential expression was identified.

|  | **Gene Name** | **# Space DE** | **Expression** |
| --- | --- | --- | --- |
| **soxS** | *pgi* | 5 | Overexpressed |
|  | *marA* | 5 | Overexpressed |
|  | *marR* | 5 | Overexpressed |
|  | *nfo* | 4 | Overexpressed |
|  | *nfsB* | 4 | Overexpressed |
|  | *waaY* | 4 | Overexpressed |
|  | *waaZ* | 4 | Overexpressed |
|  |  |  |  |
|  | ***Gene Name*** | ***# Space DE*** | ***Expression*** |
| **oxyR** | *grxA* | 6 | Underexpressed |
|  | *yhjA* | 6 | Underexpressed |
|  | *hcp* | 5 | Underexpressed |
|  | *ahpC* | 4 | Underexpressed |
|  | *dps* | 4 | Underexpressed |
|  | *ahpF* | 4 | Underexpressed |
|  | *sufA* | 4 | Underexpressed |
|  | *uxuA* | 4 | Underexpressed |
|  |  |  |  |
|  | ***Gene Name*** | ***# Space DE*** | ***Expression*** |
| **marA** | *putA* | 6 | Overexpressed |
|  | *marR* | 5 | Overexpressed |
|  | *nfo* | 4 | Overexpressed |
|  | *nfsB* | 4 | Overexpressed |
|  | *slp* | 4 | Underexpressed |
|  | *waaY* | 4 | Overexpressed |
|  | *waaZ* | 4 | Overexpressed |
